# Using Smooth Pursuit Calibration for Difficult-to-Calibrate Participants

**DOI:** 10.16910/jemr.10.4.1

**Published:** 2017-10-04

**Authors:** Pieter Blignaut

**Affiliations:** University of the Free State Bloemfontein, South Africa

**Keywords:** Calibration, Smoot pursuit

## Abstract

Although the 45-dots calibration routine of a previous study ( 2) provided very good accuracy, it requires intense mental effort and the routine proved to be unsuccessful for young children who struggle to maintain concentration. The calibration procedures that are normally used for difficult-to-calibrate participants, such as autistic children and infants, do not suffice since they are not accurate enough and the reliability of research results might be jeopardised. Smooth pursuit has been used before for calibration and is applied in this paper as an alternative routine for participants who are difficult to calibrate with conventional routines. Gaze data is captured at regular intervals and many calibration targets are generated while the eyes are following a moving target. The procedure could take anything between 30 s and 60 s to complete, but since an interesting target and/or a conscious task may be used, participants are assisted to maintain concentration. It was proven that the accuracy that can be attained through calibration with a moving target along an even horizontal path is not significantly worse than the accura-cy that can be attained with a standard method of watching dots appearing in random order. The routine was applied successfully for a group of children with ADD, ADHD and learning abilities. This result is important as it provides for easier calibration – especially in the case of participants who struggle to keep their gaze focused and stable on a stationary target for long enough.

## Introduction

Video-based eye tracking is based on the principle that near-infrared light shone onto the eyes is reflected off the different structures in the eye to create four Purkinje reflections ( 1 ). The standard way of calibrating such eye trackers is through presentation of a series of dots (or gaze targets) at known positions on the display and expect the participant to watch the dots until enough gaze data is sampled ( 3 ). While expensive commercial systems utilise a model of the eye to compute the gaze direction ( 4 ), selfassembled eye trackers use mostly polynomial expressions to map the relative position of the pupil to the corneal reflections (the so-called pupil-glint vector) to gaze coordinates. A least squares estimation is used to minimise the distances between the observed points and the actual points in the calibration grid ( 5 ).

Normally, five or nine dots are used. The more dots that are used, the better the accuracy of the system should be. Good accuracy is important when the stimuli is close to each other as in reading, where a researcher wants to determine the number of fixations on individual syllables. A procedure is described in a previous study ( 2 ) where 45 dots are displayed in a 9×5 grid. Twenty-three of the dots are used as calibration targets, while the complete set of dots is used to select the best possible regression polynomial. The dots are displayed in random order to prevent participants to pre-empt the position of the next dot and take their eyes away from a dot before the gaze was registered.

While the procedure described in Blignaut ( 2 ) is accurate with a reported average offset of 0.32°, it requires intense and prolonged concentration and participants do not always understand that they have to keep their eyes fixated on a dot until the next one appears. Unsurprisingly, the routine proved to be unsuccessful for young children with Attention Deficit Disorder (ADD), Attention Deficit Hyperactivity Disorder (ADHD) and learning disabilities. An occupational therapist using the system complained that young the children with these conditions did not understand exactly what was expected of them and some of them could not maintain concentration for the entire period.

The challenge is, therefore, to capture gaze data at as many known locations as possible, with the least possible mental effort while maintaining attention on the target. In this paper, a smooth pursuit calibration routine is proposed with a target moving across the display at a constant speed. The target could also be an animated image of something of interest to a small child, such as a butterfly or an airplane. In order to further motivate the child participant to watch the target closely, it could change colour, shape or image at varying intervals and the child could be challenged to count the number of changes.

The need for calibration and existing calibration procedures are discussed in the following section. The difficulties that are experienced with the standard routines to calibrate certain groups of participants (collectively referred to as difficult-to-calibrate (DC) participants) are highlighted and previous attempts to solve the problem are discussed. Thereafter, the presentation of a moving target with a related task is offered as a solution to capture the attention of the DC participants for long enough so that the procedure can be completed.

The evaluation of smooth pursuit calibration (SPC) is done in two phases: First, the accuracy of the approach is validated based on comparison with a standard calibration procedure using able and cooperating participants. Second, the applicability of the approach is validated for a group of early primary school children with various forms of o ne or more cognitive disorders.

The paper concludes with a discussion of the results.

## The Role of Calibration

### The need for calibration

The output from eye-tracking devices varies with individual differences in the shape or size of the eyes, such as the corneal bulge and the relationship between the eye features (pupil and corneal reflections) and the foveal region on the retina. Ethnicity, viewing angle, head pose, colour, texture, light conditions, position of the iris within the eye socket and the state of the eye (open or closed) all influence the appearance of the eye ( 4 ) and, therefore, the quality of eye-tracking data ( 6 ). In particular, the individual shapes of participant eye balls, and the varying positions of cameras and illumination require all eye-trackers to be calibrated.

### The procedure

Calibration refers to a procedure to gather data so that the coordinates of the pupil and one or more corneal reflections in the coordinate system of the eye-video can be converted to x- and y-coordinates that represent the participant’s point of regard in the stimulus space. The procedure usually consists of asking the participant to look at a number of pre-defined points at known angular positions while storing samples of the measured quantity ( 7-9 ). There is no consensus on exactly when to collect these samples, but Nyström, Andersson ( 3 ) showed that participants know better than the operator or the system when they are looking at a target.

### Mapping to point of regard

The transformation from eye-position to point of regard can be either model-based (geometric) or interpolation-based ( 4 ). With model-based gaze estimation, a model of the eye is built from the observable eye features (pupil, corneal reflection, etc.) to compute the gaze direction. In this case, calibration is not used to determine the actual gaze position but rather to record the e ye features from different angles. See Hansen and Ji ( 4 ) for a comprehensive overview of possible transformations.

Interpolation might involve, for example, a linear regression between the known data set and the corresponding raw data, using a least squares estimation to minimize the distances between the observed points and the actual points ( 5 ). Other examples of 2-dimensional interpolation schemes can be found in McConkie ( 10 ) as well as Kliegl and Olson ( 8 ) while a cascaded polynomial curve fit method is described in Sheena and Borah ( 11 ).

Theoretically, the transformation should remove any systematic error, but the limited number of calibration points that are normally used limits the accuracy that can be achieved. Typical calibration schemes require 5 or 9 pre-defined points, and rarely use more than 20 points ( 12 ).

### Auto-calibration

Huang, Kwok ( 13 ) presented an auto-calibrating system that identifies and collects gaze data unobtrusively during user interaction events since there is a likely correlation between gaze and cursor and caret locations. The procedure presented by Huang, Kwok ( 13 ) recalibrates continuously and becomes more and more accurate with additional use. They reported an average error of 2.56° which is not good but has the advantage that there is no need for an explicit calibration phase.

Swirski and Dodgson ( 14 ) describe a procedure that fits a pupil motion model to a set of eye images. Information from multiple frames is combined to build a 3D eye model that is based on assumptions on how the motion of the pupil is constrained. No calibration is needed and since the procedure is based on pupil ellipse geometry alone, it is not necessary to illuminate the eyes to create a corneal reflex. At best, a mean error of 1.68° was reported.

In summary, while auto-calibration might solve the problem of calibrating for participants who struggle to maintain concentration, it is not good enough for studies where high accuracy is needed.

## Previous Attempts to Track Difficult-to-Calibrate Participants

In the quest for a solution to calibrate young children who find it difficult to concentrate on a target for the duration of a calibration routine, one can learn from the experience of others who faced similar challenges, for example tracking infants, toddlers and children with autism.

Tracking infants and toddlers pose a challenge as it is hardly ever possible to get them to sit down long enough to focus on calibration targets. Aslin ( 15 ) mentioned that small flashing (or shrinking) targets work well with infants, but argued that accuracy is unlikely to ever be better than 1° because infants are unable to precisely and reliably fixate small stimuli. He further asserted that if 1° of accuracy is insufficient to answer a particular question, then an eye tracker should not be used for the research and alternative methods should be implemented.

Sasson and Elison ( 16 ) indicated that eye tracking of young children with autism involves unique challenges that are not present when tracking normal-developing older children or adults. They used the normal calibration routines provided by the manufacturer to track the gaze data of their participants, but used large stimuli, spanning more than 5°. Although participants find such stimuli pleasing to look at, the researcher cannot be exactly sure where the participant looked at the time of data capture. This will almost certainly result in bad accuracy that will not be feasible for tasks where high accuracy is required, such as reading.

In a study by Pierce, Conant ( 17 ), toddlers were seated on their parent’s lap in front of a Tobii T120 eye tracker and a partition separated the operator from the toddler. To obtain calibration information, toddlers were shown images of an animated cat that appeared in 9 locations on the screen. Using a software facility that superimposes the point of regard on the test image in real time, the operator observed the infant’s gaze position and head position on a secondary monitor, making note of obvious deviations from expected gaze positions. The entire process was repeated if the infant’s eyes were no longer picked up. No mention was made of the achieved accuracy, but it is reasonable to expect that the accuracy could not be better than the size of the calibration stimulus (the animated cat).

Franchak, Kretch ( 18 ) used a head-mounted eye tracker but displayed stimuli on a computer screen. A sounding target appeared at a single location within a 3×3 matrix on the monitor to induce eye movements. Calibration involved as few as 3 and as many as 9 points spread across visual space. Subjective judgement was used to determine whether fixations deviated from targets by more than about 2° and the procedure was repeated if necessary. Although the spatial accuracy is lower than that of typical desk-mounted systems, it was regarded as adequate for determining the target of fixations in natural settings. The entire process of preparing the equipment and calibrating the infant took about 15 minutes.

Corbetta, Guan ( 19 ) followed a similar procedure to calibrate an ETL-500 head-mounted eye tracker through timely coordination between one experimenter facing the child and another experimenter running the interactive calibration software of the eye tracker. The experimenter facing the child was presenting a small, visually attractive and sounding toy at one of the five predefined spatial positions. When the child was staring at the toy in that position, the experimenter running the calibration software was prompted to capture the gaze data. The researchers did not report the accuracy achieved, but one can once again assume that the accuracy could not be better than the size of the toy used as calibration stimulus.

In summary, it is clear that in an attempt to calibrate so-called difficult-to-calibrate participants, various researchers used sound and animation of larger objects as calibration targets. Furthermore, the number of calibration points is mostly limited and the accuracy that can be achieved is not expected to be better than 2°. It is also difficult to tell the actual accuracy that was obtained during a specific experimental set-up or participant recording. The need exists, therefore, for a calibration routine that is easy to execute and can be used for difficult-to-calibrate participants, yet accurate enough to provide reliable research results – especially if the experiment involves smaller or closely spaced targets.

## Smooth Pursuit Calibration

### Smooth pursuit eye movements

Smooth-pursuit eye movements are continuous, slow rotations of the eyes ( 20 ) that are used to stabilise the image of a moving object of interest on the fovea, thus maintaining high acuity ( 21, 22 ). Conscious attention is needed to maintain accurate smooth pursuit ( 23, 24 ).

Smooth pursuit gain is expressed as the ratio of smooth eye movement velocity to the velocity of a foveal target ( 25 ). If the gain is less than 1, gaze will fall behind the target to create a retinal slip that will have to be reduced by one or more "catch-up" saccades ( 26 ). According to Meyer, Lasker ( 27 ), normal subjects can follow a target with a gain of 90% up to a target velocity of 100 deg/s.

Smooth pursuit gain increases with age, especially for the first 3 months of an infant’s life ( 28, 29 ). Accardo, Pensiero ( 30 ) found that velocity gain of children aged 712 is slightly lower than that of adults.

Smooth pursuit can also be affected by attention ( 31, 32 ). More specifically, performance with smooth pursuit tasks can be dramatically improved when subjects are asked to analyse some or other changing characteristic of the target, such as reading a changing letter or number on the target ( 33 ) or pressing a button ( 34 ).

Smooth pursuit performance is also affected by stimulus background ( 35-37 ), target position ( 38 ), target velocity ( 27, 39 ), target visibility ( 40, 41 ), target direction ( 42 ) and predictability of target direction ( 43 ).

Smooth pursuit impairment and dysfunction can be linked to mental illnesses such as schizophrenia ( 33,44,45,46 ), autism ( 47 ), physical anhedonia and perceptual aberrations ( 48-50 ), Alzheimer’s disease ( 51 ) and attentiondeficit hyperactivity disorder (ADHD) ( 52 ).

### Smooth pursuit calibration in general

The concept of calibrating while a participant follows a moving target has been exploited with success in the past. Pfeuffer, Vidal ( 53 ) explains a procedure where gaze data for calibration is only sampled when the participant is attending to the target as indicated by high correlation between eye and target movement. They showed that pursuit calibration is tolerant to interruption and can be used to calibrate without participants being aware of the procedure.

Pfeuffer, Vidal ( 53 ) used a Tobii TX300 eye tracker to test their calibration procedure and collected gaze data at 60 Hz. At a target speed of 5.8°/s, it took 20 seconds to complete the target trajectory and an accuracy of just less than 0.6° were achieved. The results were compared with the 5-point calibration routine of Tobii which took 19 seconds to complete and delivered an accuracy of ≈0.7°.

Celebi, Kim ( 54 ) follows the approach of Pfeuffer, Vidal ( 53 ) but argues that an Archimedean spiral would provide better spatial coverage of the stimulus plane with little redundancy. At a linear velocity of 6.4°/s, the calibration procedure took 27 seconds during which 1600 data points were collected. Upon testing 10 healthy adults on an Eyelink 1000 eye tracker running at at 500 Hz, their approach delivered an average accuracy of 0.84° compared to 1.39° with a standard 9-point calibration procedure (which took 23 seconds to complete).

Celebi, Kim ( 54 ) stated explicitly that the goal of their smooth pursuit approach towards calibration is to improve the calibration for toddlers and children with or without developmental disabilities although they did not test the approach with such participants.

Gredebäck, Johnson ( 55 ) describes a calibration routine that makes use of a moving object to lure infants’ eyes to 2 or 5 calibration targets, but they reported accuracy according to the manufacturer’s specifications of 0.5° - a value which has been computed with a conventional calibration routine under ideal circumstances and with cooperating adult participants.

### Smooth pursuit calibration for DC participants

Participants who struggle to maintain concentration o n tedious tasks can be cognitively stimulated by indicating or counting the number of transitions of the target from one stimulus to another ( 33 ). In this study, participants were requested to follow a grey disk of 1.5° diameter on a white background (Figure 1) that contained a coloured dot (0.2°) in the centre. The dot changed colour in cycles of blue (2 seconds) and red (500 ms) and the participants were then asked to say the word "Red" aloud every time that the disk changed to red.

The target is initially displayed statically in the top left corner and the participant can be prepared as to the direction and nature of the motion that could be expected. The experimenter can initiate movement with a button as soon as the participant is ready. Three alternative trajectories were tested with the target moving along an even horizontal path (Figure 1a) , a wavy horizontal path (Figure 1b) and vertical Figure 1c. 

**Figure 1a:**
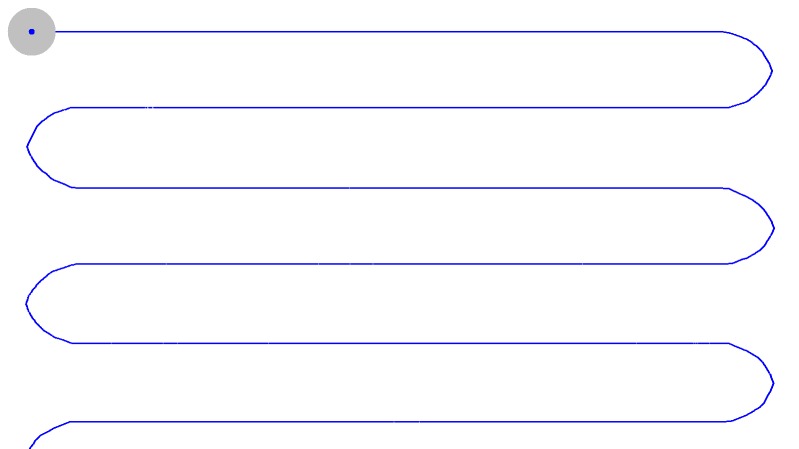
Trajectories of moving target

**Figure 1b:**
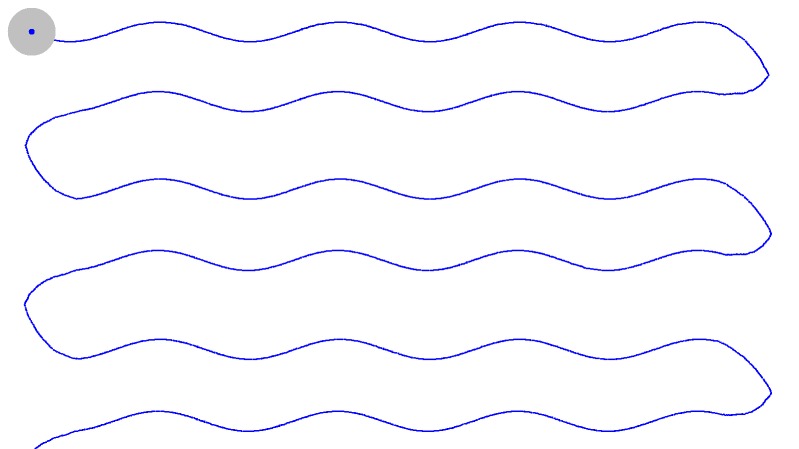
Trajectories of moving target

**Figure 1c:**
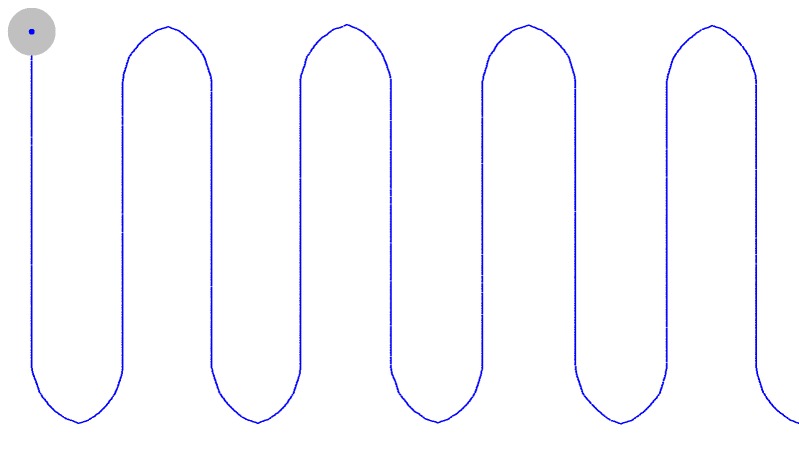
Trajectories of moving target

Depending on the speed of movement, the interval between windows and the trajectory, a large number of targets can be extracted from the continuous gaze data. In the procedure of Pfeuffer, Vidal ( 53 ), gaze data is collected whenever a participant attends to the moving target. In our approach, gaze samples (or more specifically, pupil-glint vectors for each eye) are captured for very short windows (100 ms) at intervals of 500 ms. When data is not available at a specific interval, the point is ignored. This means that, at a framerate of 200 Hz, 20 samples were recorded within a 100 ms window. At a velocity of 6.65°/s (gaze distance 700 mm, 300 px/s on a 19.5", 1600×900 screen), the samples would span 0.665° in the direction of movement.

The radius of curvature was set so that the horizontal and vertical trajectories would cover 6 and 9 distinct Y and X coordinates respectively (cf Figure 1). This resulted in 76 targets (in 38 s) being captured for both the even and wavy horizontal movements and 66 targets (in 33 s) for the vertical movement.

Since the eyes move smoothly to follow the target, it can be expected that a convex hull around the sample points would be elongated along the direction of movement. The samples within every window were sorted according to the x and y dimensions of the pupil-glint vectors and only the intersection of the centre 80% of samples around the median in each dimension are retained.

In contrast with a standard 5-point or 9-point calibration procedure where all points are needed for the regression, the multitude of points that are available with this procedure allows the removal of points where participants blinked or where their attention was distracted. All windows for which the dispersion (Max(maxX-minX, (maxYminY)) of contained samples are above 5°, are also removed. For each of the remaining windows of gaze data samples, the average location and the average pupil-glint vector are calculated.

From here on, the procedure as explained in Blignaut ( 2 ) is followed. The gaze data windows represent calibration points at known locations and are used to determine a gaze mapping polynomial set per participant. Regression coefficients are recalculated in real-time – based on a subset of calibration points in the region of the current gaze. Real-time localized corrections are done that are based on calibration targets in the same region. See Blignaut ( 2 ) for a detailed discussion of the procedure.

## Accuracy of Smooth Pursuit Calibration

In this section, the accuracy of the approach is validated based on a comparison with a standard calibration procedure using healthy and cooperating adult participants. The applicability of the approach for difficult-tocalibrate participants will be addressed in the next section.

### Equipment

For this study, an eye tracker with two infrared illuminators, 480 mm apart, and the UI-1550LE camera from IDS Imaging (https://en.ids-imaging.com) was assembled. All recordings were made at a framerate of 200 Hz.

Every frame that is captured by the eye camera was analysed and the centres of the pupils and the corneal reflections (glints) were identified. A regression-based approach was followed to map the pupil-glint vector to a point of regard in display coordinates. The regression coefficients are determined through a calibration process.

### Method

Seventeen healthy and cooperating adult participants were recruited through convenience sampling and presented with four calibration routines, namely a moving target along an even horizontal path, a moving target along a wavy horizontal path and a target moving vertically (cf Figure 1). The 45-dots routine as proposed in a previous study ( 2 ) was also presented for comparison purposes. The procedure was executed only once for every participant.

After every routine, a 7×4 grid of dots was displayed to determine the accuracy of the procedure. As for the 45 dots, the 28 dots appeared in random order to prevent participants from pre-empting the position of the next dot and prematurely look away. The regression coefficients as determined in the preceding calibration routine was used to map the gaze data to screen coordinates. The accuracy for a specific participant was calculated as the average offset between the known locations and the reported gaze coordinates across the 28 dots. The performance of a calibration routine is expressed as the average accuracy over all participants.

### Recording of calibration points

**Figure 2:**
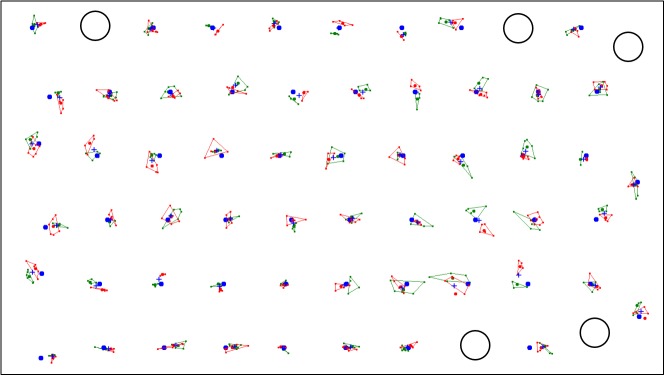
Calibration points with accompanying gaze data samples as captured with a horizontal-ly moving target. Missing points marked with circles.

Figure 2 shows the calibration points that were recorded for a specific participant while the target was moving along an even horizontal path. The mapped gaze coordinates of the sample data are enclosed by convex hulls – green for the left eye and red for the right eye.

In the example presented in Figure 2, five of the calibration windows did not contain enough sample data – probably due to blinks. Table 1 shows that for routines that involve a moving target, on average between 2 and 4 calibration windows are lost in this way. Since there are more than enough other points to be used in the subsequent regression and because the lost points are seldom at successive locations, this does not pose a problem.

**Table 1:** Average number of points (across participants) with enough samples and with mapped gaze coordinates within 1° of the target per calibration routine (SD: Standard deviation)

		Points with enough samples			Points within 1° of target			
Routine	Possible points	Avg	SD	%		Avg	SD	%
45 dots	45	45	0	100		39.3	10.0	87.3
Even hor	76	72.5	6.1	95.4		69.1	8.2	90.9
Wavy hor	76	74.1	2.9	97.5		69.7	7.1	91.6
Vertical	66	63.9	3.3	96.9		60.9	6.0	92.3

Initially, the set of calibration points was also used as validation targets and the offsets between the calibration points and the mapped gaze coordinates were calculated. All points with offsets larger than 1.0° were then removed from the set of calibration targets and the regression procedure was repeated. The remaining calibration points are shown in blue in Figure 2, while black dots indicate points that were excluded for real-time interpolation. Table 1 also shows the final number of points that was used to determine the polynomial coefficients through regression.

### Validation results

**Figure 3:**
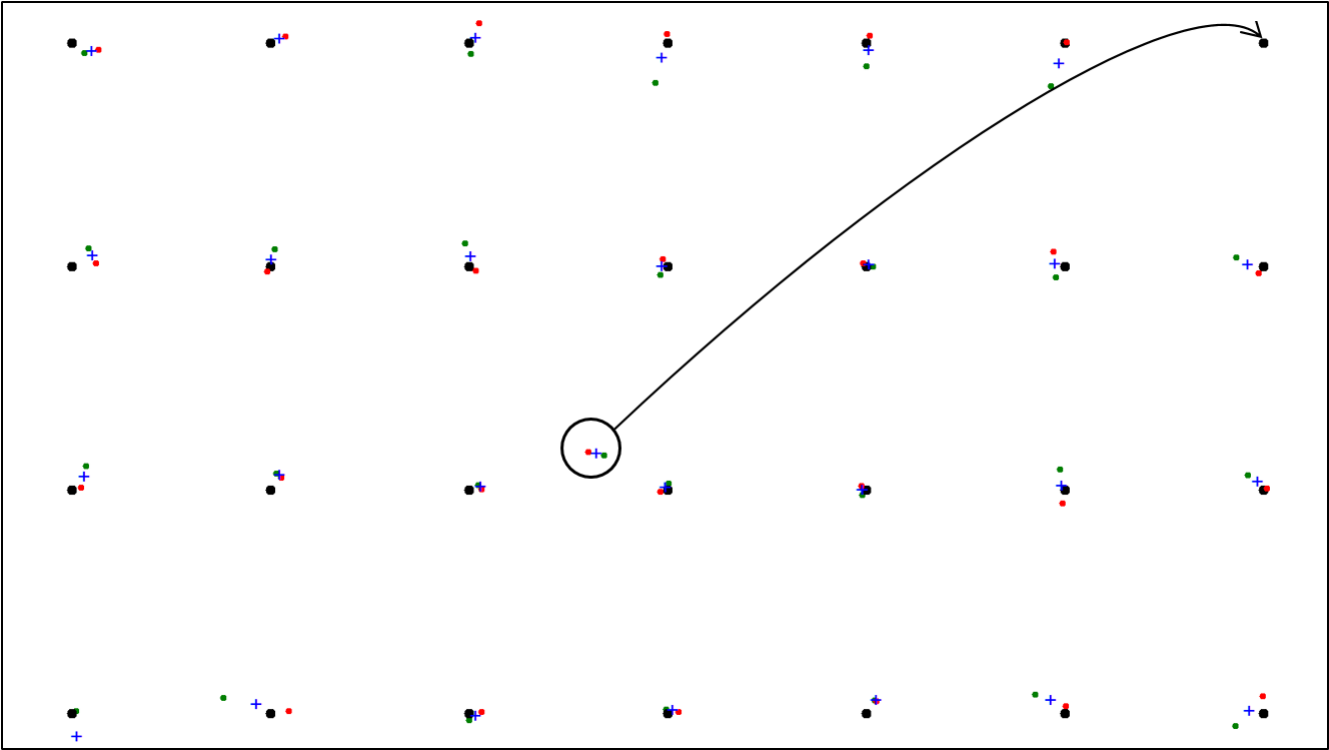
Validation points with left (green) and right (red) eye averages of samples per point. The average between eyes is indicated with a blue +. A point where the participant was distracted, is also shown.

Figure 3 shows the validation points for a calibration that was done for a moving target along an even horizon-tal path. The average of all samples within a window is shown for the left and right eyes. A + indicates the average position between the left and right eyes.

The example in Figure 3 was specifically selected to illustrate the occurrence of outliers. These outliers may occur if the participant loses concentration or is distracted by external stimuli. Sometimes (some of) the samples are captured during a blink, in which case the samples for the left and right eyes appear to be disconnected. Validation points were excluded from the calculation of average offset if the offset was larger than 3° or if the mapped gaze coordinates for the two eyes were more than 3° apart. Table 2 shows the average number of validation points that was included for each of the calibration routines. These thresholds were set large enough not to exclude valid data but small enough to ensure that unwanted gaze behaviour is excluded.

**Figure 4:**
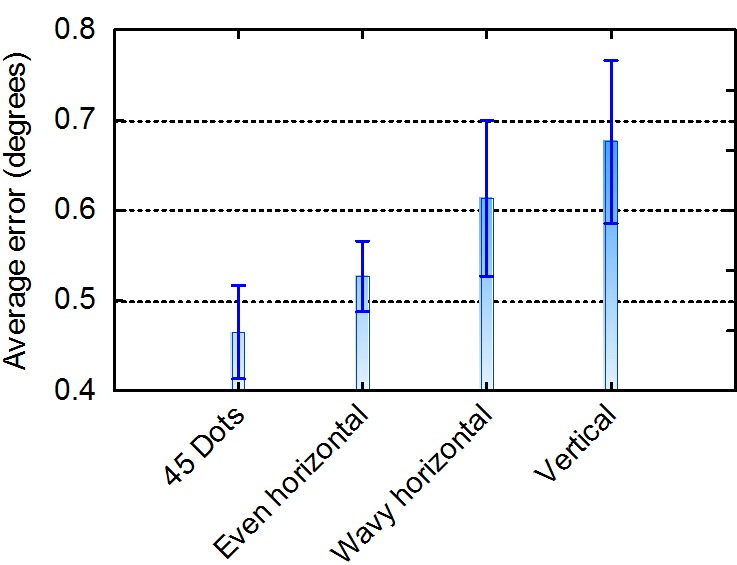
Average error over participants and validation targets for four calibration routines. The vertical bars denote the 95% confidence intervals of the means

Table 2 also shows the average error across the 28 validation points and 17 participants per calibration routine. The important column that needs to be interpreted to compare the four calibration routines is boldfaced. Figure 4 provides a visualisation of the same results. The vertical bars denote the 95% confidence intervals of the means.

**Table 2:** Average number of validation points that was included and the average error (over participants and validation targets) for each of the calibration routines. (SD: Standard deviation, SEM: Standard error of the mean)

	Number of points					Error (degrees)				
Routine	Min	Max	Avg	SD		Min	Max	Avg	SD	SEM
45 dots	14	28	25.7	4.4		0.31	0.65	0.47	0.10	0.024
Even hor	24	28	27.1	1.2		0.41	0.68	0.53	0.08	0.019
Wavy hor	19	28	26.6	2.3		0.39	1.03	0.61	0.17	0.041
Vertical	23	28	26.6	1.6		0.45	1.18	0.68	0.18	0.043

A repeated measures analysis of variance (each participant calibrated with four different routines) showed that the calibration routine has a significant (α = .001) effect on the magnitude of the error (F(3,48) = 9.74, p < .000). Table 3 shows the results of Tukey’s test for the honestly significant difference between pairs of means. The differences between the means for the 45-dots and vertical movement as well as the difference between 45-dots and horizontal movement along a wavy path were significant (α = .01). Although the accuracy for the moving target along an even horizontal path was worse than that of the 45-dots routine, it was not significantly so (α = .05).

It can, therefore, be concluded that a moving target along an even horizontal path has the potential to be used as alternative calibration routine. This will be tested in the next section with a sample of difficult-to-calibrate participants.

**Table 3:** p-Values for the significance of the difference in error between pairs of means

	Even hor	Wavy hor	Vertical
45 dots	0.465	0.005	0.000
Even hor		0.183	0.005
Wavy hor			0.457

### Applicability of Smooth Pursuit Calibration for Difficult-to-Calibrate Participants

### Equipment

The same self-assembled eye tracker was used as in the previous section.

### Method

A school for learners with special education needs were visited and all learners from Grade 1 to Grade 3 (ages 6 – 11) for which permission of the parents were obtained, were tested. The school accommodates learners who are cerebrally palsied, physically and/or learning disabled. The school has specially qualified remedial teachers as well as a multi-disciplinary support structure that includes psychologists, social workers, occupational therapists, speech therapists, physiotherapists as well as a professional nurse. The school follows the normal mainstream syllabi but there are no more than 10 learners in a class to enable teachers to provide specialised and individual attention.

Several lessons were learned in the process of capturing data. Initially, learners were requested to follow the moving target without any further instruction. It soon became evident that they struggle to maintain focus on the target for the duration of the trajectory. The target was then programmed to change colour in cycles of blue (2 seconds) and red (500 ms) and learners were instructed to call out the word "Red" whenever the target changes to red. For the procedures where dots were involved, every dot appeared in a different colour and learners were instructed to call out the colour for the dot every time.

Although the system allows moderate head movements, many learners had excessive sideways and back and forth head movements – some of which were involuntary. A chinrest was then used to maintain head position, but it caused instability of the eyes every time that the learners vocalised their response on a colour change of the target. Finally, the learners were instructed to push their foreheads against a barrier that was set such that a fixed gaze distance of 700 mm was maintained.

It was also realised that the sets of 45 dots for calibration and 28 dots for validation was too exhausting and therefore these were limited to 23 calibration targets (in rows of 5, 4, 5, 4, 5 targets each) and 15 validations targets in a grid of 5×3.

Eventually, 24 participants were tested with the final configuration of target movement, headrest and calibration sets. The number of learners per grade and condition is shown in Table 4. Note that some learners had more than one condition.

**Table 4:** Number of learners per grade and condition (ADD: Attention deficit disorder; ADHD: Attention deficit hyperactivity disorder; ASP: Asperger syndrome; DSL: Dyslexia; EPSY: Epilepsy; LD: Learning disability)

Grade	n	Age	ADD	ADHD	ASP	DSL	EPSY	LD
1	4	6.75	1	3		1	1	1
2	13	8.62	5	2	2			6
3	7	9.86		4				4

The learners were presented with a moving target along an even horizontal path (SP) (*cf* Figure 1) and the 23-dots routine. After every routine, the 5×3 grid of dots was displayed to determine the accuracy of the procedure. As was the case for the validation of accuracy with healthy adults, the dots appeared in random order to prevent learners from pre-empting the position of the next dot and prematurely look away. The performance of both the 23-dot and SP calibration routines was expressed as the average accuracy over all participants as determined through the 15 dots validation routine.

### Validation results

As for the validation of accuracy with healthy adults, validation points were excluded from the calculation of average offset if the offset was larger than 3° or if the mapped gaze coordinates for the two eyes were more than 3° apart. Table 5 shows the average number of validation points that were included for each one of the calibration routines. A repeated measures analysis of variance for the effect of calibration routine (23-dots vs SP) on the number of valid points showed that the SP routine leads to more reliable data as there are significantly less points that have to be discarded (F(1,47) = 47.7, p < .001).

Table 5 also shows the average error across the 15 validation points and 24 participants per calibration routine. A repeated measures analysis of variance for the effect of calibration routine (23-dots vs SP) on the accuracy of tracking showed that the SP routine is significantly better than a dots-based routine (F(1,47) = 12.57, p < .000) for difficult-to-calibrate participants.

**Table 5:** Average number of validation points that was included and the average error (over participants and validation targets) for each of the calibration routines. (SD: Standard deviation, SEM: Standard error of the mean)

	Number of points					Error (degrees)				
Routine	Min	Max	Avg	SD		Min	Max	Avg	SD	SEM
28 dots	2	15	10.6	3.34		0.60	2.49	1.15	0.39	0.080
Even hor	9	15	13.7	1.67		0.53	1.77	0.94	1.15	0.235

## Summary

The conventional way of calibrating remote videobased eye trackers is through presentation of a series of gaze targets at known positions while participants are expected to watch the targets. For regression-based mapping of eye features to gaze coordinates, more gaze targets normally mean better (more accurate) calibration. Unfortunately, more gaze targets also require more mental effort from participants. Through informal observations, it was realised that, although the 45 dots-routine of a previous study ( 2 ) provided very good accuracy, it expected too much mental effort for participants who struggle to maintain concentration.

Depending on the type of experiment, better accuracy might be expected than can be achieved with calibration free or auto-calibrating systems. The calibration procedures that are normally used for infants, toddlers and autistic children do also not suffice since they are not accurate enough and the reliability of research results might be jeopardised.

In this paper, the use of smooth pursuit with a target moving across the display at a constant speed, is proposed. This approach is motivated by the fact that attention to a moving target can be maintained more easily – especially if accompanied by a concurrent and related task such as analysis of some or other changing characteristic of the target ( 33, 34 ).

While the participant is following the target, gaze data is captured at regular intervals and many calibration targets are saved that can be used in subsequent regression and interpolation. Because of the abundance of points, the procedure allows the exclusion of points of dubious quality. Depending on the speed of movement and the trajectory, the procedure could take anything between 30 s and 60 s to complete.

Validation of the proposed routine was done in two phases: The accuracy of the routine was validated by comparing its performance with that of a standard calibration procedure for healthy and cooperating adults. Thereafter, the applicability of the approach for participants who are normally difficult to calibrate, is validated by applying it for a group of early primary school children with various forms of one or more cognitive disorders.

It was proven through a repeated measures, within-participants, analysis of variance that the accuracy that can be attained through calibration with a moving target along an even horizontal path is not significantly worse than the accuracy that can be attained with a standard method of watching dots. Accuracy of around the 0.5° mark were obtained for both routines for a group of seventeen adults which is comparable with the 0.6 attained by Pfeuffer, Vidal ( 53 ) and better than the 0.84° attained by Celebi, Kim ( 54 ).

For a group of young children with various forms of cognitive disorders such as ADD, ADHD and learning disabilities, smooth pursuit calibration proved to be superior to the standard routine. For this group, an average accuracy of below 1° could be achieved with SP while it was not the case with a standard routine of 28 dots. This is a significant improvement on the 1.5°-2.5° errors that can be attained by calibration-free or auto-calibrating routines such as those of Huang, Kwok ( 13 ) and Swirski and Dodgson ( 14 ).

## Future Research

Since smooth pursuit ability develops until the age of adolescence ( 30 ), one can expect that older children will benefit even more from the smooth pursuit approach. This needs to be investigated.

Furthermore, the smooth pursuit approach was tested above for children with ADD, ADHD and learning disabilities. No children with autism were tested and it remains to be seen of the approach will work for such conditions since it is known that smooth pursuit is impaired in autism and similar conditions ( 17, 47 ).
